# Active case finding to detect symptomatic and subclinical pulmonary tuberculosis disease: implementation of computer-aided detection for chest radiography in Viet Nam

**DOI:** 10.5365/wpsar.2024.15.4.1118

**Published:** 2024-10-12

**Authors:** Anh L Innes, Andres Martinez, Gia Linh Hoang, Thi Bich Phuong Nguyen, Viet Hien Vu, Tuan Ho Thanh Luu, Thi Thu Trang Le, Victoria Lebrun, Van Chinh Trieu, Nghi Do Bao Tran, Nhi Dinh, Huy Minh Pham, Van Luong Dinh, Binh Hoa Nguyen, Thi Thanh Huyen Truong, Van Cu Nguyen, Viet Nhung Nguyen, Thu Hien Mai

**Affiliations:** a FHI 360 Asia Pacific Regional Office, Bangkok, Thailand.; b FHI 360, Durham, North Carolina, United States of America.; c FHI 360 Viet Nam, Hanoi, Viet Nam.; d United States Agency for International Development/Viet Nam, Hanoi, Viet Nam.; e Viet Nam National Lung Hospital, Hanoi, Viet Nam.; f Pulmonology Department, University of Medicine and Pharmacy, Viet Nam National University, Hanoi, Viet Nam.

## Abstract

**Objective:**

In Viet Nam, tuberculosis (TB) prevalence surveys revealed that approximately 98% of individuals with pulmonary TB have TB-presumptive abnormalities on chest radiographs, while 32% have no TB symptoms. This prompted the adoption of the “Double X” strategy, which combines chest radiographs and computer-aided detection with GeneXpert testing to screen for and diagnose TB among vulnerable populations. The aim of this study was to describe demographic, clinical and radiographic characteristics of symptomatic and asymptomatic Double X participants and to assess multilabel radiographic abnormalities on chest radiographs, interpreted by computer-aided detection software, as a possible tool for detecting TB-presumptive abnormalities, particularly for subclinical TB.

**Methods:**

Double X participants with TB-presumptive chest radiographs and/or TB symptoms and known risks were referred for confirmatory GeneXpert testing. The demographic and clinical characteristics of all Double X participants and the subset with confirmed TB were summarized. Univariate and multivariable logistic regression modelling was used to evaluate associations between participant characteristics and subclinical TB and between computer-aided detection multilabel radiographic abnormalities and TB.

**Results:**

From 2020 to 2022, 96 631 participants received chest radiographs, with 67 881 (70.2%) reporting no TB symptoms. Among 1144 individuals with Xpert-confirmed TB, 51.0% were subclinical. Subclinical TB prevalence was higher in older age groups, non-smokers, those previously treated for TB and the northern region. Among 11 computer-aided detection multilabel radiographic abnormalities, fibrosis was associated with higher odds of subclinical TB.

**Discussion:**

In Viet Nam, Double X community case finding detected pulmonary TB, including subclinical TB. Computer-aided detection software may have the potential to identify subclinical TB on chest radiographs by classifying multilabel radiographic abnormalities, but further research is needed.

In 2022 alone, approximately 10.6 million people fell ill with TB globally. (*1*) Although new diagnostic tests are improving the capacity for early detection, TB remains one of the world’s deadliest infectious diseases. (*2*-*4*) While chest radiographs (CXRs) are used to screen for TB, their interpretation capacity is limited in many high-TB burden settings. Recognizing this barrier to early detection, in March 2021, the World Health Organization (WHO) endorsed the use of artificial intelligence-powered computer-aided detection (CAD) in place of human readers to interpret digital CXRs for TB among individuals aged 15 years and older. (*5*) WHO describes four models for integrating CAD into TB screening or triage algorithms. (*6*) These models differ in the way CAD is used alongside human readers to interpret CXRs. They comprise: CAD screening followed by human reading for all abnormal CXRs; CAD screening followed by human reading for all abnormal CXRs plus a proportion of normal CXRs; CAD and human reading conducted in parallel; and human reading replaced by CAD.

In recent years, the spectrum of pulmonary TB has broadened and now includes terms describing early stages of disease such as “minimal” and “subclinical” TB. (*7*-*9*) Subclinical TB is “disease due to viable *Mycobacterium tuberculosis* bacteria that does not cause clinical TB-related symptoms but causes other abnormalities that can be detected using existing radiologic or microbiologic assays.” (*7*) Furthermore, it was previously thought that TB transmission only occurred when symptoms such as cough were present, but recent studies demonstrate that people with subclinical TB disease are infectious (*10*) and exhale *M. tuberculosis* bacteria (evidenced by face-mask sampling). (*11*) Prevalence surveys from 23 African and Asian countries show that 36–80% of individuals with TB disease have no TB symptoms. (*12*) Neglecting to diagnose and cure subclinical TB disease is thus a barrier to ending TB.

Screening of any TB symptom (cough, haemoptysis, fever, night sweats or weight loss) has an estimated 71% sensitivity for identifying TB disease. (*6*) CXR screening using TB-presumptive abnormalities significantly improves case detection, in particular of subclinical TB, increasing sensitivity to 85%. (*6*) While not as accurate as chest computed tomography (CT) imaging for detecting subclinical and incipient TB, (*13*-*15*) CXRs remain the most pragmatic, readily available radiographic option for TB screening and triage in high-TB burden settings, especially when coupled with CAD technologies. However, to date, few studies have assessed the extent to which CAD products improve the accuracy of CXR screening for subclinical TB in routine programme implementation.

Viet Nam’s second national TB prevalence survey, conducted in 2017–2018, found a bacteriologically-confirmed TB prevalence of 322 cases per 100 000 persons; among individuals with confirmed TB disease, 97.7% had CXR abnormalities suggesting TB, 57.9% reported cough for 2 or more weeks and 32.1% had no TB symptoms. (*16*) These findings led Viet Nam’s National Tuberculosis Program (NTP) to implement a “Double X” (2X) strategy to diagnose TB among symptomatic and asymptomatic TB-vulnerable populations, which used CXR to identify individuals for confirmatory diagnostic testing with GeneXpert (Xpert; Cepheid, Sunnyvale, CA, United States of America). From 2020 to 2022, CAD was integrated into NTP’s 2X community case-finding strategy. The aim of this study was to describe the demographic, clinical and radiographic characteristics of symptomatic and asymptomatic 2X participants, including those diagnosed with TB. CAD-scored radiographic abnormalities were also assessed to determine whether they were associated with Xpert-confirmed TB disease, both overall and separately for symptomatic and subclinical TB.

## Methods

### Setting

This study was conducted as part of routine programmatic implementation from March 2020 to December 2022. Annual 2X active case finding community campaigns were conducted in eight provinces comprising An Giang, Can Tho, Dong Nai, Dong Thap, Nghe An, Tay Ninh, Tien Giang and Thai Binh, which were selected for being representative of Viet Nam’s three regions and for their baseline TB notification rates. Collectively, the eight provinces accounted for approximately 20% of the country’s notified TB cases. The 2X community campaigns ranged in duration from 4 to 18 days and evaluated between 100 and 440 individuals daily.

### Community TB screening algorithms

The 2X community participants comprised two categories of TB-vulnerable populations. The first category was household contacts of adults diagnosed with pulmonary TB disease (with or without bacteriological confirmation) within 2 years of the start of the 2X campaign. Contacts were persons who had lived, slept (1 night per week) or stayed (1 hour per day, 5 days per week) in the same house with the index patient for 3 months before diagnosis. The second category of TB-vulnerable populations included individuals who were aged 60 years and older (the age category defined as “elderly” according to Vietnamese law (*17*)), had a diagnosis of diabetes, or were smokers (any smoking history), regular alcohol users (daily) or malnourished (low body mass index), as well as those with pulmonary or other chronic diseases, a history of prior treatment for TB disease or living with HIV. Medical history was self-reported. TB symptoms (fever, cough of any duration, weight loss or night sweats) were documented but not required for CXR evaluation. For participants with “TB-presumptive” CXRs, sputum specimens were collected on site for Xpert testing. Physicians also referred participants for Xpert testing if they had normal CXRs but positive screens for TB symptoms and/or TB risk factors, based on participant interview during campaign intake.

### CXR interpretation by physicians

Posterior-anterior digital CXR images (Vikomed, Hanoi, Viet Nam) were obtained in mobile CXR vans and interpreted in the van by provincial-level radiologists who had access to each participant’s name, age and brief medical history including TB symptoms and risk factors. CXRs reviewed by physicians were interpreted as “TB-negative” or “TB-presumptive.”

### CAD analysis

Offline CAD analysis with qXR (*18*) version 3.0 (Qure.ai, Mumbai, India) was performed using qBoxes installed in mobile CXR vans. Each CXR DICOM (Digital Imaging and Communications in Medicine) image was given a qXR TB abnormality score, which ranged from 0.00 to 1.00, with higher values indicating more abnormal CXRs. The manufacturer’s pre-set threshold for TB interpreted a qXR score ≥ 0.50 as TB-presumptive and < 0.50 as TB-negative. qXR employs convolutional neural networks-based algorithms that are able to perform “multilabel” classification of other, non-TB radiographic abnormalities, including blunted costophrenic angle, calcification, cardiomegaly, cavity, consolidation, fibrosis, hilar lymphadenopathy, nodule, opacity, pleural effusion and pneumothorax. (*19*-*21*) This feature extends the capability of qXR beyond providing simple binary TB-presumptive and TB-negative results. (*22*, *23*) Thresholds for the 11 multilabel, non-TB radiographic abnormalities analysed in this study were pre-set by the manufacturer and did not change during the study (2020–2022).

**Fig. 1:**
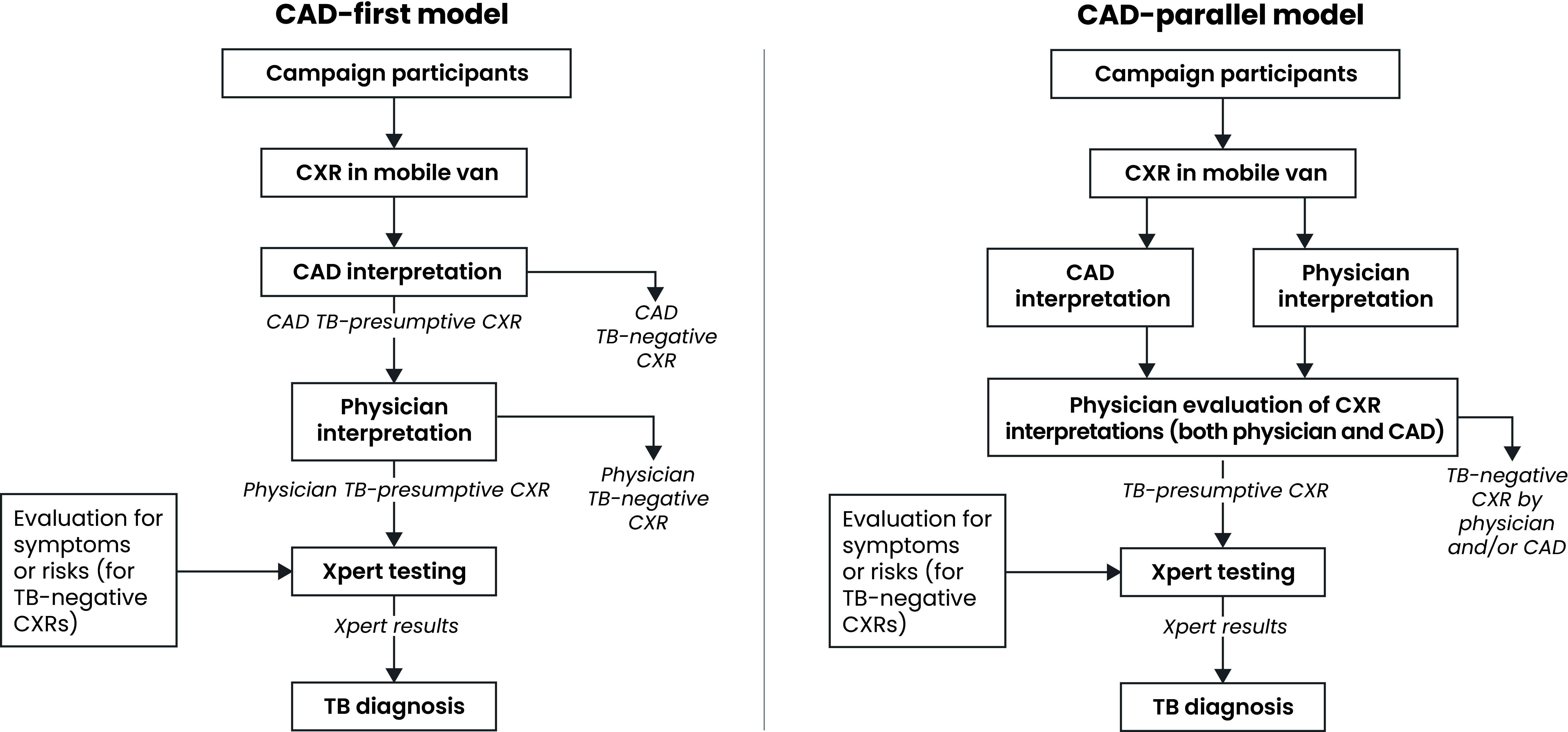
Models employed for CAD-CXR interpretation for TB in Viet Nam^a,b^

To select CAD TB thresholds for 2X implementation, we conducted a retrospective qXR analysis of CXRs from 2020 community campaigns, which showed that threshold scores from 0.40 to 0.60 resulted in the most consistent case-finding yields across provinces. Thresholds in this range were employed in 2X campaigns in 2021 onwards. In terms of the choice of CAD integration model, a priori, there was no preference for a “CAD-first” (software interprets CXRs first and only those rated as CAD TB-presumptive are read by physicians) or a “CAD-parallel” model (CXRs are interpreted by both CAD and on-site physicians; **Fig. 1**), and both models were employed in 2021 – CAD-first at three sites and CAD-parallel at two sites. For the CAD-first model, we selected a qXR ≥ 0.40 TB threshold, which is lower and thus more sensitive than the manufacturer’s pre-set threshold (≥ 0.50), to reduce the risk for missing potential cases. Conversely, a qXR ≥ 0.60 TB threshold was selected for the “CAD-parallel” model; this is higher than the pre-set ≥ 0.50 threshold and was selected to increase CAD specificity for the parallel model and reduce the risk of false positives. To standardize methods in 2022, all CXRs were processed according to the CAD-first model (and qXR threshold ≥ 0.40), which was simpler to implement than CAD-parallel integration in mobile CXR vans. qXR interpreted CXRs from all 2X participants who were aged 6 years and older.

[**insert** Figure 1]

### Diagnostic confirmation with Xpert testing

Participants referred for Xpert testing produced a single-spot specimen that was analysed on site or in a nearby facility with Xpert capacity (Xpert MTB/RIF or Xpert Ultra). Symptomatic TB disease was defined as Xpert-confirmed TB in individuals with any TB symptom (fever, cough of any duration, weight loss or night sweats), and subclinical TB disease was defined as Xpert-confirmed TB with no TB symptoms.

### Statistical analysis

Demographic and clinical characteristics of the 2X study participants were summarized and compared across the study years using the χ^2^ test (categorical variables) and ANOVA (continuous variables). The characteristics of the subsets of participants with symptomatic and subclinical Xpert-confirmed TB were also compared using the χ^2^ test. Univariate and multivariable logistic regression modelling explored which, if any, characteristics (region, sex, age group, smoking status, prior treatment for TB, diabetes, alcohol use, malnutrition) were associated with subclinical Xpert-confirmed TB. We also reported the prevalence of the 11 qXR multilabel, non-TB radiographic abnormalities for each year of our study (2020–2022). Finally, we used univariate and multivariable Firth logistic regression to determine which of the 11 multilabel, non-TB radiographic abnormalities were associated with Xpert-confirmed TB (overall) and which were associated with subclinical Xpert-confirmed TB. The Firth logistic regression model uses a penalized log-likelihood to handle separation, which prevents cases from being dropped and enables use of the full sample. All models were fitted on data from 2020, 2021, 2022 and all years combined. Data were analysed using STATA 18 (Stata Corp; College Station, TX, United States).

## Results

We retrospectively analysed 51 441 CXRs from 2020 and used real-time CAD to analyse 17 078 CXRs in 2021 and 28 112 CXRs in 2022. Participants’ demographic and clinical characteristics differed across the three years (**Table 1**). The proportion of participants aged 60 years and older was lower in 2022 (40.5%) compared to 2020 (47.1%) and 2021 (46.7%). Females outnumbered males in all years, with the lowest proportion of males (41.1%) recorded in 2020. Participants in 2021 were the least symptomatic, with only 16.8% reporting any symptom compared to 31.8% in 2020 and 33.9% in 2022; cough was the most frequently reported symptom. Across all years, a total of 15 278 participants had CXRs that were rated as TB-presumptive (15.8%), among whom 14 024 (91.8%) underwent Xpert testing and 1254 (8.2%) dropped out. Additionally, 1200 who had TB-negative CXRs but presented with TB symptoms and/or risk factors underwent Xpert testing, for a total of 15 224 (92.1% TB-presumptive CXRs, 7.9% TB-negative CXRs) (**Table 1**). Across the study period, Xpert positivity averaged 7.5% (1144/15 224). Xpert positivity was lower among those with TB-negative CXRs (2.9%) and among those with subclinical TB (5.9%).

**Table 1:** Participant characteristics for 2X community case finding, 2020–2022

Characteristics	2020–2022 (*n* = 96 631)	2020 (*n* = 51 441)	2021 (*n* = 17 078)	2022 (*n* = 28 112)
**Region**				
**North (%)**	**14 624 (15.1)**	**11 825 (23.0)**	**2799 (16.4)**	**–**
**Central (%)**	**6072 (6.3)**	**3675 (7.1)**	**2397 (14.0)**	**–**
**South (%)**	**75 935 (78.6)**	**35 941 (69.9)**	**11 882 (69.6)**	**28 112 (100)**
**Age^a^**				
**Mean (SD)**	**54.32 (18.17)**	**54.88 (18.14)**	**54.34 (19.05)**	**53.31 (17.62)**
**Median (IQR)**	**58 (45–67)**	**58 (46–67)**	**58 (45–67)**	**56 (43–66)**
**Sex**				
**Female (%)**	**54 535 (56.6)**	**30 320 (58.9)**	**8886 (52.3)**	**15 329 (54.9)**
**Male (%)**	**41 829 (43.4)**	**21 114 (41.1)**	**8116 (47.7)**	**12 599 (45.1)**
**Screening group**				
**Household contacts (%)**	**20 996 (21.7)**	**12 587 (24.5)**	**4575 (26.8)**	**3834 (13.6)**
**Other vulnerable populations (%)**	**75 627 (78.3)**	**38 846 (75.5)**	**12 503 (73.2)**	**24 278 (86.4)**
**Specific vulnerable populations^b^**				
**Elderly^c^ (≥ 60 years) (%)**	**43 481 (45.1)**	**24 153 (47.1)**	**7967 (46.7)**	**11 361 (40.5)**
**Prior TB treatment (%)**	**9218 (9.5)**	**5114 (9.9)**	**2074 (12.1)**	**2030 (7.2)**
**Smoker (%)**	**11 805 (12.2)**	**5957 (11.6)**	**1826 (10.7)**	**4022 (14.3)**
**Alcohol use disorder (%)**	**2706 (2.8)**	**1253 (2.4)**	**445 (2.6)**	**1008 (3.6)**
**Malnutrition (%)**	**1001 (1.0)**	**652 (1.3)**	**116 (0.7)**	**233 (0.8)**
**Diabetes (%)**	**9268 (9.6)**	**5169 (10.0)**	**1275 (7.5)**	**2824 (10.0)**
**Hypertension (%)**	**33 639 (34.8)**	**17 365 (33.8)**	**5404 (31.6)**	**10 870 (38.7)**
**Asthma (%)**	**4067 (4.2)**	**1304 (2.5)**	**856 (5.0)**	**1907 (6.8)**
**Chronic obstructive pulmonary disease (%)**	**1677 (1.7)**	**772 (1.5)**	**620 (3.6)**	**285 (1.0)**
**Symptoms**				
**Cough of any duration (%)**	**25 447 (26.3)**	**14 499 (28.2)**	**2639 (15.5)**	**8309 (29.6)**
**Fever (%)**	**1580 (1.6)**	**1197 (2.3)**	**55 (0.3)**	**328 (1.2)**
**Night sweats (%)**	**2624 (2.7)**	**1462 (2.8)**	**98 (0.6)**	**1064 (3.8)**
**Weight loss (%)**	**3947 (4.1)**	**2524 (4.9)**	**301 (1.8)**	**1122 (4.0)**
**Any symptom (%)**	**28 750 (29.8)**	**16 333 (31.8)**	**2875 (16.8)**	**9542 (33.9)**
**CXR and Xpert results**				
**CAD TB-presumptive CXR (%)**	**ND**	**6934 (13.5)**	**2892 (16.9)**	**5033 (17.9)**
**Physician TB-presumptive CXR (%)**	**15 278 (15.8)**	**7406 (14.4)**	**3789 (22.2)**	**4083 (14.5)**
**Xpert testing (rate,%)** ^d^	**15 224 (15.8)**	**7205 (14.0)**	**3722 (21.8)**	**4297 (15.3)**
**Xpert positivity^e^**				
**Overall (%)**	**1144 (7.5)**	**620 (8.6)**	**194 (5.2)**	**330 (7.7)**
**TB-negative CXR (%)**	**35 (2.9)**	**20 (4.4)**	**10 (2.8)**	**5 (1.3)**
**Subclinical TB (%)**	**584 (5.9)**	**302 (6.8)**	**130 (4.4)**	**152 (5.9)**
**Symptomatic TB (%)**	**560 (10.7)**	**318 (11.5)**	**64 (8.7)**	**178 (10.2)**
**Xpert-confirmed TB yield overall per 100 000 CXR**	**1184**	**1205**	**1136**	**1174**

2X: Double X; *N*: number; SD: standard deviation; IQR: interquartile range; TB: tuberculosis; CXR: chest radiography; Xpert: GeneXpert; CAD: computer-aided detection; ND: not determined due to differing CAD models/thresholds each year.

^a^ Some age data were missing: *n* = 149 (2020); *n* = 23 (2021); *n* = 62 (2022).

^b^ Characteristics for people living with HIV are not summarized due to small sample size (*n* = 65 for all years).

^c^ Age cut-off for “elderly” (aged ≥ 60 years) was defined using the Viet Nam Law for the Elderly. (*17*)

^d^ Number of participants who underwent Xpert testing (*n* = 15 224) as a percentage of the total number of participants with CXRs (*n* = 96 631). Note that this number includes 1200 participants with physician TB-negative CXRs who also underwent Xpert testing. The decision to offer Xpert testing for these participants was at the discretion of the on-site physician based on an assessment of symptoms and risk factors.

^e^ Number of participants with a positive Xpert test result (*n* = 1144) as a percentage of the number of participants who underwent confirmatory Xpert testing (*n* = 15 224).

Among the 1144 individuals who were diagnosed with Xpert-confirmed TB disease during 2020–2022, around half had subclinical TB (51%). However, this proportion differed by year, geographical region, age group, prior TB treatment, smoking status, alcohol use and malnutrition (**Table 2**; **Supplementary Table 1**). Subclinical TB prevalence was higher in the northern region than in the central and southern regions (72.5%, 36.7% and 49.8%, respectively). Subclinical TB prevalence was higher among older age groups and in those with a history of TB treatment than those without (55.2% versus 48.3%). Subclinical TB prevalence was lower in smokers than non-smokers (43.2% versus 54.6%), those with alcohol use disorders than those without (38.2% versus 51.9%), and those with malnutrition than those without (20.0% versus 51.5%). In multivariable logistic regression models, residing in the northern region (adjusted odds ratio [aOR]: 2.37; 95% confidence interval [CI]: 1.42–3.96) and prior treatment for TB (aOR: 1.36; 95% CI: 1.05–1.75) were associated with higher odds of subclinical TB disease. Age groups 30–39 and 40–49 years (aOR: 0.50; 95% CI: 0.30–0.85 and aOR: 0.55; 95% CI: 0.36–0.84, respectively), smoking (aOR: 0.68; 95% CI: 0.52–0.90) and malnutrition (aOR: 0.28; 95% CI: 0.08–0.99) were associated with lower odds of subclinical TB. Neither sex nor self-reported diabetes was associated with subclinical TB.

**Table 2:** Demographic and clinical characteristics of individuals with Xpert-confirmed TB disease, comparing subclinical and symptomatic disease, and associations with subclinical TB, 2020–2022^a^

Characteristics	Subclinical TB	Symptomatic TB	Total	OR (95% CI) (*n* = 1 144)	aOR (95% CI) (*n* = 1 141)
**Positive Xpert result**	**584 (51.0)**	**560 (49.0)**	**1144**	**–**	**–**
**Physician TB-presumptive CXR**	**561**	**548**	**1109**	**–**	**–**
**Physician TB-negative CXR**	**23**	**12**	**35**	**–**	**–**
**Region**					
**North (%)**	**58 (72.5)**	**22 (27.5)**	**80**	2.66* (1.60–4.41)	2.37** (1.42–3.96)
**Central (%)**	**11 (36.7)**	**19 (63.3)**	**30**	**0.58 (0.27–1.24)**	**0.47 (0.22–1.02)**
**South (%)**	**515 (49.8)**	**519 (50.2)**	**1034**	**Reference**	**Reference**
**Sex**					
**Male (%)**	**484 (50.8)**	**468 (49.2)**	**952**	**0.95 (0.70–1.30)**	**1.09 (0.78–1.54)**
**Female (%)**	**100 (52.1)**	**92 (47.9)**	**192**	**Reference**	**Reference**
**Age groupb**					
**0–19 (%)**	**5 (45.5)**	**6 (54.5)**	**11**	**0.67 (0.20–2.20)**	**0.63 (0.19–2.09)**
**20–29 (%)**	**9 (45.0)**	**11 (55.0)**	**20**	**0.65 (0.27–1.60)**	**0.68 (0.27–1.69)**
**30–39 (%)**	**27 (38.6)**	**43 (61.4)**	**70**	0.50** (0.30–0.83)	0.50*** (0.30–0.85)
**40–49 (%)**	**48 (42.1)**	**66 (57.9)**	**114**	0.58** (0.39–0.87)	0.55** (0.36–0.84)
**50–59 (%)**	**156 (49.1)**	**162 (50.9)**	**318**	**0.77 (0.59–1.01)**	**0.81 (0.61–1.07)**
** ≥ 60 (%)**	**338 (55.6)**	**270 (44.4)**	**608**	**Reference**	**Reference**
**Prior treatment for TB**					
**No (%)**	**336 (48.3)**	**359 (51.7)**	**695**	**Reference**	**Reference**
**Yes (%)**	**248 (55.2)**	**201 (44.8)**	**449**	1.32*** (1.04–1.67)	1.36*** (1.05–1.75)
**Smoker**					
**No (%)**	**431 (54.6)**	**359 (45.4)**	**790**	**Reference**	**Reference**
**Yes (%)**	**153 (43.2)**	**201 (56.8)**	**354**	0.63* (0.49–0.82)	0.68** (0.52–0.90)
**Diabetes**					
**No (%)**	**517 (51.3)**	**491 (48.7)**	**1008**	**Reference**	**Reference**
**Yes (%)**	**67 (49.3)**	**69 (50.7)**	**136**	**0.92 (0.64–1.32)**	**0.91 (0.63–1.32)**
**Alcohol use disorder**					
**No (%)**	**558 (51.9)**	**518 (48.1)**	**1076**	**Reference**	**Reference**
**Yes (%)**	**26 (38.2)**	**42 (61.8)**	**68**	0.57*** (0.35–0.95)	**0.79 (0.46–1.34)**
**Malnutrition**					
**No (%)**	**581 (51.5)**	**548 (48.5)**	**1129**	**Reference**	**Reference**
**Yes (%)**	**3 (20.0)**	**12 (80.0)**	**15**	0.24*** (0.07–0.84)	0.28*** (0.08–0.99)

aOR: adjusted odds ratio; CXR: chest radiograph; OR: odds ratio; TB: tuberculosis; Xpert: GeneXpert.

* *P* < 0.001; ** *P* < 0.01; *** *P* < 0.05.

^a^ All characteristics shown in this table were included as predictors in the multivariable logistic regression model. We opted for the most parsimonious model and thus did not include hypertension, asthma and chronic obstructive pulmonary disease as predictors since they did not change the included predictors’ statistical significance for association with the outcome. HIV was not included as a predictor due to small sample size (*n* = 65 for all years).

^b^ Logistic regression models that include age groups have a sample of *n* = 1141 (3 cases missing information on age).

Among those with Xpert-confirmed TB, the most frequently classified CAD radiographic abnormalities were consolidation, fibrosis, nodule and opacity (**Table 3**). In adjusted analyses, cavity, consolidation, fibrosis, nodule and opacity were significantly associated with higher odds of Xpert-confirmed TB. Fibrosis was also associated with higher odds of subclinical TB (aOR: 1.77; 95% CI: 1.10–2.85), while consolidation was associated with lower odds of subclinical TB (aOR: 0.71; 95% CI: 0.52–0.97) for all years combined (**Table 4**).

**Table 3:** Distribution of CAD multilabel, non-TB radiographic abnormalities among CXRs with Xpert-confirmed TB, 2020–2022

Radiographic abnormality	qXR threshold	2020 *n* = 620 (%)	2021 *n* = 194 (%)	2022 *n* = 330 (%)
**Blunted costophrenic angle**	**0.80**	**12.3**	**9.3**	**14.5**
**Calcification**	**0.85**	**–**	**33.5**	**40.6**
**Cardiomegaly**	**0.85**	**1.9**	**0.5**	**2.7**
**Cavity**	**0.90**	**33.4**	**42.8**	**42.4**
**Consolidation**	**0.50**	**71.0**	**77.3**	**79.1**
**Fibrosis**	**0.70**	**90.5**	**92.3**	**90.3**
**Hilar lymphadenopathy**	**0.85**	**1.6**	**3.1**	**3.3**
**Nodule**	**0.50**	**86.9**	**86.6**	**90.0**
**Opacity**	**0.50**	**96.9**	**97.9**	**99.1**
**Pleural effusion**	**0.75**	**12.6**	**12.9**	**13.9**
**Pneumothorax**	**NA**	**–**	**1.0**	**0.6**

CAD: computer-aided detection; CXR: chest radiography; NA: not available; qXR: Qure.ai CAD software; TB: tuberculosis; Xpert: GeneXpert.

**Table 4:** Summary of associations between CAD radiographic abnormalities and Xpert-confirmed/subclinical Xpert-confirmed TB disease, 2020–2022

Radiographic abnormality	aOR^a^ (95% CI) for Xpert-confirmed TB disease			
	All years (*N*^b^ = 15 224)	2020 (*n*^b^ = 7 205)	2021 (*n*^b^ = 3 722)	2022 (*n*^b^ = 4 297)
**Blunted costophrenic angle**	0.72 (0.54–0.95)*	**0.95 (0.64–1.40)**	0.36 (0.17–0.75)**	**0.66 (0.41–1.08)**
**Calcification**	0.63 (0.53–0.75)***	**0.33 (0.02–5.94)**	**0.80 (0.57–1.13)**	0.60 (0.46–0.78)***
**Cardiomegaly**	**0.79 (0.51–1.23)**	**0.84 (0.46–1.52)**	**0.57 (0.11–3.00)**	**0.82 (0.41–1.65)**
**Cavity**	1.71 (1.47–1.98)***	**1.23 (1.00–1.51)**	2.80 (1.97–3.97)***	2.18 (1.65–2.88)***
**Consolidation**	4.40 (3.76–5.15)***	3.79 (3.07–4.67)***	5.27 (3.53–7.85)***	5.14 (3.80–6.96)***
**Fibrosis**	1.38 (1.08–1.77)*	1.64 (1.16–2.31)**	**1.39 (0.73–2.67)**	**1.01 (0.66–1.55)**
**Hilar lymphadenopathy**	**0.95 (0.62–1.43)**	**1.03 (0.52–2.03)**	**0.82 (0.35–1.92)**	**1.12 (0.58–2.15)**
**Nodule**	2.06 (1.67–2.53)***	1.98 (1.50–2.62)***	1.65 (1.01–2.68)*	2.26 (1.51–3.38)***
**Opacity**	2.51 (1.59–3.95)***	2.27 (1.30–3.98)**	**2.95 (0.97–9.01)**	**2.90 (0.93–9.04)**
**Pleural effusion**	**0.89 (0.68–1.18)**	**0.74 (0.50–1.09)**	**1.29 (0.66–2.53)**	**1.01 (0.61–1.66)**
**Pneumothorax**	**0.49 (0.18–1.32)**	**4.53 (0.18–111.69)**	**0.73 (0.17–3.07)**	**0.35 (0.09–1.33)**

Radiographic abnormality	aOR^a^ (95% CI) for subclinical Xpert-confirmed TB disease			
	All years (*N*^c^ = 1 144)	2020 (*n*^c^ = 620)	2021 (*n*^c^ = 194)	2022 (*n*^c^ = 330)
**Blunted costophrenic angle**	**1.06 (0.62–1.79)**	**1.35 (0.65–2.82)**	**0.35 (0.08–1.57)**	**1.38 (0.56–3.40)**
**Calcification**	**1.26 (0.92–1.74)**	**NA^d^**	**1.22 (0.62–2.40)**	**1.27 (0.78–2.06)**
**Cardiomegaly**	**0.61 (0.25–1.44)**	**0.77 (0.25–2.39)**	**1.28 (0.05–33.28)**	**0.35 (0.08–1.57)**
**Cavity**	**0.85 (0.65–1.12)**	**0.80 (0.56–1.16)**	**1.41 (0.72–2.78)**	**0.67 (0.40–1.13)**
**Consolidation**	0.71 (0.52–0.97)*	0.58 (0.39–0.88)*	**0.91 (0.40–2.08)**	**0.97 (0.53–1.77)**
**Fibrosis**	1.77 (1.10–2.85)*	2.15 (1.11–4.15)*	**0.69 (0.15–3.19)**	**1.63 (0.71–3.73)**
**Hilar lymphadenopathy**	**1.00 (0.47–2.14)**	**0.80 (0.23–2.74)**	**0.96 (0.19–4.98)**	**1.15 (0.34–3.88)**
**Nodule**	**0.93 (0.62–1.42)**	**1.30 (0.73–2.30)**	**0.99 (0.34–2.89)**	**0.55 (0.25–1.23)**
**Opacity**	**0.49 (0.20–1.22)**	**0.62 (0.21–1.83)**	**0.29 (0.01–7.09)**	**0.12 (0.01–2.62)**
**Pleural effusion**	**0.65 (0.39–1.11)**	**0.57 (0.27–1.19)**	**0.97 (0.25–3.78)**	**0.65 (0.25–1.69)**
**Pneumothorax**	**1.55 (0.25–9.48)**	**NA^d^**	**0.45 (0.03–7.17)**	**1.89 (0.18–19.99)**

aOR: adjusted odds ratios; CAD: computer-aided detection; CI: confidence interval; NA: not available; TB: tuberculosis; Xpert: GeneXpert.

* *P* < 0.05; ** *P* < 0.01; *** *P* < 0.001.

^a^ All 11 multilabel, non-TB radiographic abnormalities, if available, were included as predictors in the multivariable logistic regression models.

^b^ Number of individuals with Xpert results.

^c^ Number of individuals with Xpert-confirmed TB disease.

^d^ Point estimates are not available because there were no CXRs with calcification or pneumothorax in 2020.

## Discussion

This study describes the Viet Nam NTP’s 2X community-based active case-finding strategy that effectively diagnosed TB among symptomatic and asymptomatic TB-vulnerable populations during 2020–2022. Of the 96 631 individuals who were targeted by 2X campaigns and screened with CXRs, 15 224 underwent Xpert testing, which was predominantly for TB-presumptive CXRs (14 024; 92.1%), and 1144 individuals were diagnosed with Xpert-confirmed TB, of whom 584 (51.0%) had subclinical TB disease. A CAD radiographic classification of fibrosis was found to be a good predictor of subclinical TB disease.

Our study provided several insights into the distribution of symptomatic versus subclinical TB disease among 2X participants. While the total number of Xpert-confirmed TB cases was lower in 2021, coinciding with Viet Nam’s most severe phase of the COVID-19 pandemic, (*24*) the proportion of participants diagnosed with subclinical TB in 2021 was higher than in other years. The reasons for this are likely multifactorial, potentially including increased stigma around respiratory diseases during the height of the COVID-19 pandemic, resulting in underreporting of TB symptoms. Also, individuals with respiratory symptoms may have been preferentially triaged to COVID-19 evaluation, leaving a higher proportion of individuals without symptoms to participate in 2X campaigns. Pandemic lockdowns in 2021 may have additionally delayed care-seeking, possibly leading to more severe – and symptomatic – TB disease being diagnosed in 2022.

We also noted that 2X campaigns in Viet Nam’s northern region detected higher proportions of subclinical TB disease than southern campaigns. This could be related to the relatively low TB prevalence in the north, especially compared with the south where some of the highest TB prevalences in the country have been recorded. (*16*, *25*) This pattern has been found in other countries, including Cambodia, China and India. (*12*) Furthermore, we observed a greater prevalence of subclinical TB among older 2X participants (≥ 60 years). This finding is in contrast with a study conducted in Republic of Korea, which found that age < 65 years was associated with subclinical TB disease. (*26*) However, the two studies are not directly comparable due to differences in the definition of subclinical TB; the Korean study defined subclinical TB as “radiographic or microbiologic results consistent with TB among individuals without clinical symptoms,” whereas ours relied solely on Xpert confirmation. Our findings regarding smoking also differ from Viet Nam’s TB prevalence survey results, which suggest that current smoking is associated with both symptomatic and subclinical TB disease. (*27*) In contrast, in our 2X population, smoking was only associated with lower odds for subclinical TB; differences in study design most likely explain the discrepancies between our study and prevalence survey results.

Fibrosis was the only CAD multilabel radiographic abnormality that was associated with higher odds of subclinical TB disease among 2X participants. A common sequela of pulmonary TB, (*28*) fibrotic lesions tend to progress and regress repeatedly and thus represent a dynamic risk. (*29*) Nevertheless, there is some evidence to suggest that the presence of fibrosis may be prognostic for TB disease; one study showed that fibrosis or infiltrates on ^18^F-FDG PET/CT can identify subclinical TB that is likely to progress to symptomatic TB disease among people living with HIV. (*14*) Others have shown that fibrotic lesions are associated with an increased risk for progression to TB disease among individuals with TB infection. (*5*, *30*) For 2X participants, consolidation (which develops when air-filled spaces in the lungs become fluid-filled) (*31*) was associated with lower odds of subclinical TB. This is not surprising, since fluid occupying air-filled spaces normally causes respiratory symptoms.

Interest in the use of chest radiography for active case finding, particularly subclinical TB, has increased in recent years; (*13*, *14*, *32*-*35*) multiple studies have been conducted in a variety of settings (high- and low-TB burden) and differing study populations (in terms of age structure or HIV status). Studies have also compared the accuracy of CXRs versus CT scans and CXR field reading versus expert reading for identifying subclinical TB abnormalities. According to one such study in a low-TB burden country, cavitation, extensive parenchymal abnormalities and endobronchial spread were more frequently missed on CXRs than on CT scans. (*13*) Another study found that cavitation and upper-lobe parenchymal abnormalities were more likely to be missed by CXR field readers than expert readers. (*33*) In our setting, while cavitation was associated with higher odds of Xpert-confirmed TB, we found no evidence of an association with subclinical TB. Using qXR’s pre-set threshold for cavitation of ≥ 0.90, we detected cavities in 33.4–42.8% of CXRs with Xpert-confirmed TB (depending on the year). It is possible that had we employed a lower, more sensitive threshold, we might have detected more cavitation and/or observed significant differences in cavitation between symptomatic and subclinical TB. Further limiting our analysis was the qXR output, which did not include an abnormality score for cavitation.

Deep learning-based CAD classification of multilabel radiographic abnormalities on CXR has demonstrated variable diagnostic accuracy for TB. An early version of qXR image classifiers matched human expert annotations for four radiographic abnormalities in drug-resistant TB CXRs. (*20*) Although qXR 2.0s discriminatory power for classifying specific chest abnormalities, measured against radiologists’ interpretations, proved to be variable, (*21*) qXR 3.0 found significant associations between upper-lobe cavitation and TB disease among diabetics. (*36*) Radiographic abnormalities classified by Lunit INSIGHT version 3.1.0.0 (Seoul, Republic of Korea) were associated with culture-confirmed TB disease but had limited sensitivity using the manufacturer’s pre-set thresholds. (*37*) Other convolutional neural networks-based algorithms reportedly classified TB-related radiographic abnormalities accurately. (*23*, *38*) Taken together, these studies suggest that CAD for CXRs may yet have value beyond providing simple binary results (TB-presumptive versus TB-negative). To date, however, no product has the proven capacity to accurately identify subclinical TB abnormalities. More research is needed to determine if CAD can improve CXR accuracy for detecting subtle lesions of early, subclinical TB disease. Clarification is needed from manufacturers on how multilabel radiographic abnormalities factor into the CAD TB threshold deep-learning algorithms. One way forward might be to focus on subclinical radiographic abnormalities that are detected on CT and PET/CT scans but are missed on CXR. For example, it might be possible to evaluate whether calibration of the fibrosis threshold improves the accuracy of CXR detection of subclinical TB disease. Cavitation, although not associated with subclinical TB in our population (possibly due to the high qXR ≥ 0.90 threshold), is another candidate for a similar evaluation. Of note, CXRs cannot detect metabolic activity in radiographic lesions; some lesions may thus exhibit the same radiographic appearance on CXRs, whether they are active or inactive, and regardless of the CAD threshold accuracy for TB or multilabel abnormalities.

In this study, the CAD-first and CAD-parallel integration models supported CXR interpretation and Xpert referral decisions along the 2X community workflow. The CAD-first model decreased physicians’ workloads by limiting the number of CXRs for them to interpret to those rated as TB-presumptive; programmatic implementation in other settings has shown similar benefits. (*34*, *39*) The CAD-first model works well in high-TB burden settings where clinical evaluation, CXR interpretation, Xpert referral decisions and sputum collection are conducted in one site – for example, community campaigns that use mobile CXR vans or ultraportable CXR units as one-stop shops. CAD-parallel interpretation was difficult to implement with fidelity in the mobile vans, since in our protocol the physicians were not blinded to the CAD result; thus, their CXR reading may have been influenced by the CAD result, even though, ideally, they should have been independent. CAD-first integration was selected for the Viet Nam community setting, while CAD-parallel integration was selected for facility-based 2X case finding. (*40*)

Our study had limitations. We conducted Xpert testing only in participants with TB-presumptive CXRs or TB symptoms or risk; we did not carry out systematic diagnostic testing in participants with normal CXRs. Not all participants with TB-presumptive CXRs underwent Xpert testing; however, this proportion was relatively small (8.2%) and thus unlikely to have significantly biased our findings. The CAD model and TB-presumptive threshold varied from 2020 to 2022, a timeframe also affected by COVID-19. Together, these factors limited comparisons of CXR results and Xpert yield across years. Therefore, in this report, we prioritized analyses of the multilabel, non-TB radiographic abnormalities, each of which had its own threshold that did not change from 2020 to 2022 and was, in theory, less affected by the CAD TB threshold. The changing CAD TB thresholds may still affect interpretation of multilabel radiographic abnormalities, especially for year-to-year comparisons.

## Conclusions

Double X TB case finding detected a high proportion of subclinical TB disease among TB-vulnerable populations in Viet Nam’s communities. While there is a clear role for CAD as a tool to aid the interpretation of digital CXRs in screening programmes for TB disease, further research is needed to determine whether CAD can improve CXR identification of subclinical TB using multilabel, non-TB radiographic abnormalities.
